# Possible Targets of Pan-Coronavirus Antiviral Strategies for Emerging or Re-Emerging Coronaviruses

**DOI:** 10.3390/microorganisms9071479

**Published:** 2021-07-10

**Authors:** Xue Li, Liying Zhang, Si Chen, Hongsheng Ouyang, Linzhu Ren

**Affiliations:** Key Laboratory for Zoonoses Research, College of Animal Sciences, Ministry of Education, Jilin University, 5333 Xi’An Road, Changchun 130062, China; Lix9916@mails.jlu.edu.cn (X.L.); zhangliy@jlu.edu.cn (L.Z.); chensi_1024@163.com (S.C.); ouyh@jlu.edu.cn (H.O.)

**Keywords:** pan-coronavirus, target, inhibitor, vaccine, epitope

## Abstract

Severe Acute Respiratory Syndrome Coronavirus-2 (SARS-CoV-2), which caused Coronaviruses Disease 2019 (COVID-19) and a worldwide pandemic, is the seventh human coronavirus that has been cross-transmitted from animals to humans. It can be predicted that with continuous contact between humans and animals, more viruses will spread from animals to humans. Therefore, it is imperative to develop universal coronavirus or pan-coronavirus vaccines or drugs against the next coronavirus pandemic. However, a suitable target is critical for developing pan-coronavirus antivirals against emerging or re-emerging coronaviruses. In this review, we discuss the latest progress of possible targets of pan-coronavirus antiviral strategies for emerging or re-emerging coronaviruses, including targets for pan-coronavirus inhibitors and vaccines, which will provide prospects for the current and future research and treatment of the disease.

## 1. Introduction

To date, seven coronaviruses have been identified in the human population, including human coronavirus (HCoV)-229E, -OC43, -NL63, and -HKU1, Severe Acute Respiratory Syndrome Coronavirus (SARS-CoV), Middle East Respiratory Syndrome Coronavirus (MERS-CoV), and Severe Acute Respiratory Syndrome Coronavirus-2 (SARS-CoV-2), which cross-transmitted from animals (bat, bovine, mouse, or other animals) to human [1]. Moreover, coronaviruses have a wide host spectrum, which was verified in numerous cell lines or animals [2,3]. For example, the SARS-CoV-2 spike could bind ACE2 (Angiotensin-converting enzyme 2) orthologs from 44 domestic animals, pets, livestock, and animals in zoos and aquaria, and initiate viral entry [3]. These results indicate that the potential spillover events of other coronaviruses from animals to humans, especially bat-derived SARS-related coronaviruses (SARSr-CoVs), are increasing, which may cause more pandemics in the future [1,4,5]. Therefore, many researchers suggest developing universal coronavirus vaccines or drugs against the next coronavirus pandemic [4,6]. We fully agree with this proposal, because effective universal vaccines or pan-coronaviruses inhibitors are promising for emerging or re-emerging coronavirus epidemics, which has attracted many groups to participate in designing universal vaccines and inhibitors for decades. However, whether for influenza, AIDS, or COVID-19, it is a challenging task to achieve this goal.

During past decades, many attempts have been made on the universal influenza vaccine, but less progress has been obtained due to three main reasons. First, because of the low conservation of virus antigens in the same viral family, especially key antigenic determinants, few conserved protective cross-reactive antigens have been identified. Second, the continuous mutations of the coronavirus lead to the immune evasion of the viruses and reduce the efficacy of existing vaccines or universal vaccines, which have also been identified in the SARS-CoV-2 variants of concern (VOCs), including Alpha (B.1.1.7), Beta (B.1.351), Gamma (P.1), and Delta (B.1.617.2) [7,8,9,10]. Notably, the Delta variant SARS-CoV-2, which is characterized by spike mutations T19R, G142D, Δ157-158, L452R, T478K, D614G, P681R, and D950N, is rapidly outcompeting other VOCs and has become the dominant variant in past months [8,11]. Third, it takes a long time for the vaccine development, clinical trial, and authorized application of the vaccine. Excitingly, with the rapid development of artificial intelligence and vaccine technology, as well as the joint efforts and close cooperation of scientists all over the world, modern vaccinology may be accelerated, and the universal vaccine may be realized in the future.

**Figure 1:**
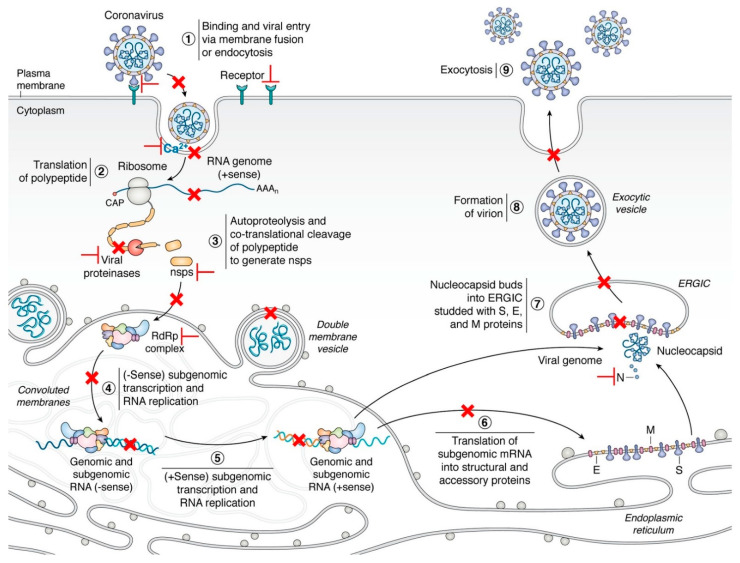
The life cycle of coronaviruses and possible targets for antivirals [18]. Key steps representing attractive antiviral targets are highlighted in red. Inhibitors can target virus fusion (especially the HR1, HR2, or 6-HB of the S2 subunit, Ca^2+^ channel), viral proteases (M^pro^ or PL^pro^), ORF1b and NSPs, replication/transcription complex (including RdRp, NSP14, and 16), viral genomic synthesis, and host factors and pathways (such as regulators of cholesterol metabolism, host proteases, HMGB1, SWI/SNF chromatin remodeling complex, cyclophilins, and immunophilins). Vaccines are designed by targeting multiple epitopes of the viral RBD, S2 subunit, as well as cross-reactive viral epitopes (in the S and ORF1ab) of B cell, CD4^+^ and CD8^+^ T cells. Reprinted with permission from ref. [18]. Copyright © 2020 Hartenian et al.

Contrary to the universal vaccine, it is scientifically feasible to develop pan-coronavirus inhibitors based on the genome-scale comprehensive analysis of virus genome and host factors using in silico virtual screening and molecular docking. Until now, numerous drugs or peptides have been identified as promising pan-inhibitors of SARS-CoV-2 and other human coronaviruses [12,13,14,15,16,17]. In this review, we discuss the latest progress of possible targets of pan-coronavirus antiviral strategies against emerging or re-emerging coronaviruses (Figure 1), which will provide prospects for the current and future research and treatment of the disease.

## 2. Targets for Pan-Coronavirus Inhibitors

During infection, the life cycle of coronaviruses can be mainly divided into nine steps, including (1) binding and viral entry via membrane fusion or endocytosis; (2) translation of polypeptide; (3) autoproteolysis and co-translational cleavage of the polypeptide to generate non-structural proteins (NSPs); (4) negative sense (-sense) subgenomic transcription and RNA replication; (5) positive sense (+sense) subgenomic transcription and RNA replication; (6) translation of subgenomic mRNA into structural and accessory proteins; (7) nucleocapsid (N) buds into ERGIC (endoplasmic reticulum-Golgi intermediate compartment) embedded with S (spike protein), E (envelope protein), and M (membrane protein) proteins; (8) formation of the virion; and (9) exocytosis [18,19]. Therefore, pan-coronavirus inhibitors are designed or repurposed to target proteins involved in these key steps, especially in the early stage of the infection (Table 1).

### 2.1. Binding and Fusion

Coronaviral spike-receptor binding and membrane fusion is the first step for virus infection, which anchors the virus on the cell surface, and initiates the virus entry [18,66]. The spike is a glycoprotein, which can be split into two subunits, S1 and S2, by host proteases [66]. The S1 subunit participates in binding with the cellular receptor, while S2 involves membrane fusion. Moreover, the functional domains signal sequence (SS), NTD (N-terminal domain), RBD (receptor-binding domain), SD1 (subdomains 1), and SD2 (subdomains 2) locate in the S1 subunit, while domains FP (fusion peptide), HR1 (heptad repeat 1), CH (central helix), CD (connector domain), HR2 (heptad repeat 2), and CT (C-terminal domain) are the main domains of the S2 subunit [18,66]. During infection, the spike interacts with the cell surface receptor, such as human aminopeptidase N (APN) for HCoV-229E, angiotensin-converting enzyme 2 (ACE2) for HCoV-NL63, SARS-CoV and SARS-CoV-2, and dipeptidyl peptidase 4 (DPP4) for MERS-CoV, via the RBD in the form of the trimer, followed by the cleavage of the spike by host proteases, such as the cell surface serine protease (TMPRSS2), furin, endosomal cathepsins L, and/or lysosomal proteases, at the boundary of the S1/S2 and S2′ site [18]. Then, the S2 subunit is activated and undergoes conformation changes, the FP domain inserts into the cell membrane, and the trimeric HR1 is surrounded by the HR2 trimer forming a six-helix bundle (6-HB), and thus leading to fusion of the viral and cellular membranes and viral entry [18,19,67].

Sequence analysis indicates that the fusion domain and fusion peptide of the S2 subunit shows a higher identity than the S1 subunit in all the coronaviruses [13,68]. The 6-HB structure is conserved and is essential for the membrane fusion and entry of coronaviruses [17]. Highly conservative hydrophobic interactions between HR1 and HR2, especially some polar and electrostatic interactions, are well preserved in different HCoVs [67]. The amino acid mutation in the HR1 domain of SARS-CoV-2 may be related to the enhanced interaction with the HR2 domain [15]. Therefore, viral HB and host proteases were selected as targets for pan-coronavirus inhibitors in recent research.

It was reported that the lipopeptides targeting membrane fusion between the virus and host cell membrane exhibited a significant antiviral effect against the direct contact transmission of SARS-CoV-2 during 24 h co-housing with infected animals via daily intranasal administration [69]. Notably, the dimeric cholesterol-conjugated peptide [SARS_HRC_-PEG_4_]_2_-chol can completely prevent virus transmission, which shows a stronger antiviral effect than other dimeric and monomeric lipid-tagged SARS-CoV-2 inhibitory peptides [69]. Lipopeptides, EK1 and EK1C4, based on the HR2 domain of HCoV-OC43, exhibit highly inhibitory activity against HCoVs (HCoV-229E, HCoV-NL63, HCoV-OC43, SARS-CoV, and MERS-CoV) and SARSr-CoVs (SARS-CoV-2 and three SARSr-CoVs, Rs3367, WIV1, SHC014) by embedding in the hydrophobic groove formed by two HR1 helices [15,17,20]. Lipopeptide EK1 can properly bind to the hydrophobic groove and interacts with HR1 via conserved hydrophobic interactions, and, thus, forming a stable 6-HB-like structure with both short α-HCoV and long β-HCoV HR1s, inhibiting the interaction between the HR1 trimer and the HR2 trimer, and blocking the viral 6-HB formation of HR1 and HR2 [17,20]. Furthermore, lipopeptide EK1C4 is generated by conjugated cholesterol on the C-terminal of EK1, which showed increased antiviral activities against all the tested HCoVs and SARSr-CoVs by targeting the HR1 domain, with about 149- to 241-fold that of the EK1 [15,17]. Moreover, the half-life of EK1C4 is longer than that of EK1 [15]. EK1C4 can effectively protect mice from infection by intranasal administration before or after the HCoV-OC43 attack, which indicates that EK1C4 can be used to prevent and treat infections caused by SARS-CoV-2 and other emerging coronaviruses [15]. Moreover, another lipopeptide, EK1V1, generated by adding cholesterol to the EK1, exhibits significantly improved antiviral activity against human coronaviruses, HIV-1, HIV-2, and SIV (simian immunodeficiency virus) [70], which indicated that lipopeptide EK1 and its derivatives are promising virus fusion inhibitors with broad-spectrum antivirals.

Meanwhile, griffithsin (GRFT), a lectin isolated from the red alga *Griffithsia sp*, can inhibit SARS-CoV-2 infection by targeting the glycosylation sites in the S1 subunit of SARS-CoV-2 S protein [71]. It is worth noting that the combination of GRFT and EK1 shows an effective synergistic effect against SARS-CoV-2 infection and has great potential in the prevention and treatment of COVID-19 due to the different targets and mechanisms of the two antiviral agents [71]. Moreover, clofazimine, an anti-leprosy drug, can efficiently antagonize coronaviruses including SARS-CoV-2, MERS-CoV, SARS-CoV, HCoV-229E, and HCoV-NL63 via inhibiting viral spike-cell fusion and viral helicase activity with negligible cytotoxicity [16]. Clofazimine can also enhance the antiviral transcriptional profile of the cell, indicating that the agent possesses pan-coronaviral inhibitory activities [16]. In addition, two clinically approved drugs, Itraconazole (ITZ) and Estradiol benzoate (EB), can inhibit coronaviruses’ entry by interacting with the HR1 or 6-HB in the S2 subunit of the S protein [21].

The coronavirus FP (fusion peptide) can be divided into two parts, the FP1 and FP2 domain, which can induce membrane ordering by directly interacting with calcium cations (Ca^2+^) [23]. Therefore, Ca^2+^ is identified as a critical factor for CoVs’ membrane fusion [22,23,24]. Recent reports showed that natural products, bis-benzylisoquinoline alkaloids, such as neferine, can block host calcium channels and, thus, inhibit Ca^2+^-mediated membrane fusion and virus entry [24,25], which can be used as pan-coronaviruses entry inhibitors.

These results further confirmed that inhibitors targeting conservative HR1, HR2, or 6-HB, as well as the membrane fusion, have the potential to be developed as fusion inhibitors of pan-coronavirus.

### 2.2. Nonstructural Protein (NSP)

Nonstructural proteins (NSPs) of coronaviruses are encoded by two open reading frames (ORF), ORF1a and ORF1b. The ORF1a encodes NSP1 to 11, while ORF1b encodes NSP12 to 16 [18,44]. During translation, polypeptide pp1a was translated from ORF1a and is cleaved into NSP 1 to 11 by the chymotrypsin-like cysteine protease (viral main protease, M^pro^, or 3CL^pro^) and the Papain-Like protease (PL^pro^). The longer polypeptide pp1ab was produced by ORF1a and ORF1b via ribosome frameshifting between the two ORFs and is proteolytically processed into NSP1 to 10, and NSP12 to 16 by the viral M^pro^ and the PL^pro^ [18].

#### 2.2.1. Viral Proteases

Among 16 nonstructural proteins, at least two kinds of proteases are identified, papain-like proteases (PL1^pro^ and PL2^pro^) are encoded by NSP3, and chymotrypsin-like cysteine protease (viral main protease, M^pro^, or 3CL^pro^) is produced by NSP5 [18]. NSP3 is the largest nonstructural protein of CoVs, with multi-domains, including ten consensus domains, ubiquitin-like domain 1 (Ubl1), Glu-rich acidic domain (hypervariable region), macrodomain (Mac, X domain), ubiquitin-like domain 2 (Ubl2), PL2^pro^, NSP3 ectodomain (3Ecto or zinc-finger domain), Y1, CoV-Y, two transmembrane regions TM1 and TM2, and several variable domains [26]. NSP3 plays various roles in virus infection. It can cleave viral polypeptide by its papain-like protease activity, and inhibit host immune responses, and support virus survival by the posttranslational modification of the host protein and itself [18,26].

Apart from polypeptide cleaving, the viral main protease can also inhibit type I IFN signaling [27,28]. Notably, the M^pro^ substrate-binding pocket is conserved in coronaviruses [13]. Therefore, Bepridil, an antianginal medicine, is effective against SARS-CoV-2 by inhibiting the viral M^pro^ in highly permissive mammalian cell lines [29]. Two M^pro^ inhibitors containing bicycloproline from Boceprevir or Telaprevir showed excellent antiviral activity in cell-based assays and transgenic mouse models [30]. The non-covalent inhibitor, ML188, exhibits potential binding activities to the M^pro^ of SARS-CoV and SARS-CoV-2, which can be used as a scaffold for a non-covalent pan-coronavirus inhibitor [31].

These results suggest that these two viral proteases (M^pro^ or 3CL^pro^) are promising targets for small-molecular antivirals or peptides against SARS-CoV-2 and other coronavirus infections.

#### 2.2.2. Replication/Transcription Complex

Another suitable target for pan-coronavirus inhibitors is the replication/transcription complex (replisome, RTC) composed of nonstructural proteins. The replisome consists of at least eight NSPs, including NSP7–10, 12–14, and 16. NSP12 (RNA-dependent RNA polymerase, RdRp), two processivity cofactor factors (NSP7 and 8), and two exonucleases (NSP14 and its cofactor NSP10) constitute the core replicase. Moreover, NSP13 is a 5′ to 3′ RNA helicase, NSP9 is a single-strand nucleic acid-binding protein, and NSP16 is a 2′-O-Methyltransferase.

Among the NSPs related to replisome, RdRp is the most promising antiviral target in RNA viruses, which has been widely used to evaluate the inhibition efficiency of numerous inhibitors in coronaviruses in vivo and in vitro [32,33,34,35,36]. The antisense oligonucleotides targeting the structural elements and the FDA-approved drugs inhibiting the viral RNA binding proteins can dramatically reduce SARS-CoV-2 infection in human cells [37]. As reported, lycorine, a phenanthridine amaryllidaceae alkaloid isolated from *Lycoris radiata* (*L’Hér.*), can efficiently inhibit MERS-CoV, SARS-CoV, and SARS-CoV-2 infections with IC_50_ values of 2.123 ± 0.053, 1.021 ± 0.025, and 0.878 ± 0.022 μM, respectively [38]. Lycorine can interact with SARS-CoV-2 RdRp on the Asp623, Asn691, and Ser759 residues through hydrogen bonds, with a higher binding affinity (−6.2 kcal/mol) than that of Remdesivir (−4.7 kcal/mol) [38]. Moreover, coronavirus RdRp, especially SARS-CoV-2 RdRp, shows a low fidelity of nucleotide insertion, which can insert nucleotide analogs into the nascent RNA, resulting in the lethal mutagenesis of the virus genome or termination of the polymerase extension [39,40,41], indicating that the nucleotide analogs are promising candidates for a pan-coronavirus inhibitor. However, numerous nucleotide analogs, such as Remdesivir and Favipiravir, have been reported to have an antiviral effect on SARS-CoV-2 in vitro [35], but none of them are effective in vivo. Therefore, there is an urgent need to further evaluate and design or modify nucleotide analogs for emerging or re-emerging coronavirus epidemics.

Moreover, the RdRp of SARS-CoV-2 and SARS-CoV contains zinc ions in two highly conserved metal-binding motifs, H295-C301-C306-C310 and C487-H642-C645-C646 [42,43]. A recent report showed that NSP12 (a catalytic subunit of RdRp) can ligate two iron-sulfur (Fe-S) metal cofactors ([Fe_4_S_4_] clusters) in the zinc centers, which are essential for replication and interaction with the viral helicase (NSP13) [43]. However, the oxidation of the [Fe_4_S_4_] clusters by the stable nitroxide TEMPOL inhibits the RdRp activity, and blocks SARS-CoV-2 replication in vitro [43], suggesting the [Fe_4_S_4_] clusters can be used as targets for therapy of COVID-19 as well as pan-coronavirus inhibitors.

Furthermore, programmatic translation frameshifting (PRF) at −1 of the ORF1b is conservative in all the coronaviruses and is necessary for the synthesis of viral RdRp and downstream viral NSPs [44,46,47]. During transcription and translation, a pseudoknot formed by three stems (stem 0, 1, and 2) on the nascent viral RNA interacts with the ribosome and 18S rRNA, causing the ribosome to pause at −1 frameshifting [44]. Meanwhile, a stop codon near the frameshifting site enhances the chances of pseudoknot refolding [44]. Thus, it is expected to develop drugs or siRNAs that interfere with the “frameshifting” and inhibit virus replication [46,48], as the inhibitory effect of the ligand on the −1 PRF is not easily evaded by mutations of the viral −1 PRF pseudoknot [48]. As Bhatt reported, merafloxacin is an effective candidate to inhibit the frameshifting, which leads to the decreases in the SARS-CoV-2 titer by 3–4 orders of magnitude, with an IC_50_ of 4.3 μM and no cellular toxicity [44]. This result was further confirmed by another group, who have shown that frameshift inhibition by merafloxacin is effective on mutations within the pseudoknot region of SARS-CoV-2 and other betacoronaviruses [45].

In addition to the inhibition of RdRp, drug hits should be evaluated for resistance to exoribonuclease (ExoN) and methyltransferase activities. NSP14 acts as (guanine-N7)-methyltransferase (N7-MTase) that catalyzes viral mRNA capping, and 3’-to-5’ proofreading ExoN that removes mis-incorporated nucleotides from the 3′ end of the nascent RNA [49,72], which are critical for virus replication and transcription [49,72]. During the capping, Cap(0)-RTC is composed of NSP12 nidovirus RdRp-associated nucleotidyltransferase (NiRAN), NSP9, NSP14, and NSP10 [72]. Therefore, NSP14 can be used as a promising antiviral target for pan-coronavirus.

Moreover, NSP16 can be activated by binding with viral NSP10 and participates in immune evasion by mimicking its human homolog, CMTr1 [50]. After activation, the NSP16 complex methylates mRNA, enhances the translation efficiency, and down-regulates the activities of RIG-I and MDA5 [18,50]. Further research identified a conserved cryptic pocket formed between β3 and β4 of viral NSP16 in SARS-CoV, SARS-CoV-2, and MERS-CoV [50]. The pocket is the critical domain for binding substrates (S-adenosylmethionine and RNA) and NSP10, suggesting the pocket site is a potential target for a pan-coronavirus inhibitor [50].

#### 2.2.3. NSP1

NSP1 is the first viral protein that cleaved from the ORF1a polyprotein of α- and β-coronaviruses by viral PL^pro^ [51]. The NSP1 sequences of different CoVs are highly divergent, but their functions are similar [51]. As reported, the viral NSP1 can inhibit cellular translation by interacting with ribosomal subunits with high affinity (especially 40S ribosomal subunit complexes) and/or inducing the degradation of the host mRNA [51,52,53,54]. Recent reports showed that the C-terminal of viral NSP1 inserted into the mRNA entry channel of the host ribosome complexes, and thereby prevented the host mRNA’s entry [52,53].

Conversely, NSP1 can also interact with the stem-loop (SL) region, especially the SL1 hairpin, of the viral 5′untranslated region (UTR), promote the escape of viral mRNAs from the NSP1-mediated translation inhibition, and facilitate the recognition and binding of viral mRNAs and host ribosome, thus enhancing the virus’ infection [51,52,53,54].

These results suggest that the viral NSP1 can be used as a therapeutic target for pan-coronavirus inhibitor and the SL1 hairpin on the viral mRNAs 5′UTR is a promising target for silencing with siRNA or sgRNA.

### 2.3. Host Proteins

Virus infection requires many host factors to participate, and there are also many host antiviral proteins to inhibit the virus infection by direct or indirect interaction. Therefore, the systematic identification of the host factors needed for coronavirus infection is an urgent task at present.

Recently, high-coverage, genome-scale screenings based on CRISPR-Cas9 genetic screening libraries were established and used to uncover interactomes including the host factors and pathways shared by coronaviruses [55,56,57]. Hoffmann et al. found that glycosaminoglycan biosynthesis, sterol regulatory element-binding protein (SREBP) signaling, bone morphogenetic protein (BMP) signaling, and glycosylphosphatidylinositol biosynthesis are potential targetable factors for pan-coronaviruses inhibitors [55,56]. In particular, host protein transmembrane protein 41B (TMEM41B) is necessary for infection of SARS-CoV-2, as well as seasonal coronaviruses HCoV-OC43, -NL63, and -229E [55]. Furthermore, TMEM41B and vacuole membrane protein 1 (VMP1) are scramblases that regulate the distribution of cholesterol and phosphatidylserine [73]. Further analysis showed that the regulators of cholesterol homeostasis, including sterol regulatory element-binding protein 2(SREBP 2), SREBP cleavage-activating protein (SCAP), membrane-bound transcription factor site 1 protease (MBTPS1), MBTPS2, Niemann–Pick intracellular cholesterol transporter 2 (NPC2), and secretion associated ras-related GTPase 1A (SAR1A), play key roles in coronaviruses’ infection [56,74]. Moreover, coronaviruses require cholesterol, for viral entry, pathological syncytia formation, and pathogenesis [58,59,60], and 25-hydrocholesterol (25HC), which is converted from cholesterol by cholesterol 25-hydroxylase (CH25H), broadly inhibits the virus-cell membrane fusion of human coronaviruses by depleting membrane cholesterol [61,62]. Therefore, targeting the key host factors of lipid metabolism, which involve membrane fusion, endolysosomal acidification, and cholesterol accumulation (such as SCAP, TMEM41B), may be a potential therapeutic strategy for pan-coronavirus. Notably, phospholipidosis is a common mechanism of antiviral activity of many repurposed drugs, which should be considered when selecting drugs [75].

Another group found that host protease Cathepsin L is specific for the SARS lineage and MERS-CoV, while viral receptors ACE2 and DPP4 are specific for both SARS-lineage viruses and MERS-CoV [57]. Furthermore, high-mobility group box 1 protein (HMGB1) is necessary for the entry of SARS lineage (SARS-CoV-2, SARS-CoV, and NL63) by regulating ACE2 expression, whereas several members of the SWI/SNF chromatin remodeling complex are pro-viral for coronaviruses, suggesting the SWI/SNF complex can be used as a pan-coronavirus target [57]. Moreover, interferon-stimulated lymphocyte antigen 6 complex locus E (LY6E) has also been identified as an anti-pan-coronavirus molecule, which can effectively inhibit multiple coronaviruses (including SARS-CoV-2, SARS-CoV, and MERS-CoV) from entering the cell by disturbing the virus-cell membrane fusion [57,63].

Pfefferle and colleagues identified that cyclophilins and immunophilins (such as PPIA, PPIB, PPIH, PPIG, FKBP1A, and FKBP1B) can interact with the NSP1 of multiple coronaviruses and modulate the Calcineurin/NFAT pathway to activate immune cells and immune responses [64,65]. On the contrary, cyclophilins (CypA/PPIA) inhibitors, such as cyclosporine A (CspA), FK506, CsD Alisporivir, NIM811, and their immunosuppressive derivatives can efficiently block the replication of CoVs of all genera, including SARS-CoV, human CoV-229E and -NL-63, feline CoV, and avian infectious bronchitis virus [64,65], indicating cyclophilin can be used as targets for broad-spectrum CoV inhibitors against coronaviruses.

## 3. Targets for Pan-Coronavirus Vaccines

Vaccines play an effective role in controlling the pandemic. However, under the pressure of host selections (including gene editing and immune response) [76], viral mutations in the prevalent strains can weaken the recognition mediated by the monoclonal antibodies and polyclonal human serum [77,78]. For example, SARS-CoV-2 undergoes continuous mutations and may finally exist as more toxic and/or more infectious variants [79], which have been circulating in Britain (B.1.1.7), South Africa (B.1.351), Brazil (P.1), India (B.1.617), and other countries recently. Furthermore, SARS-CoV-2 isolated from an immunocompromised person contains 57% and 38% mutations in the spike and its RBD, respectively [80]. Excitingly, several vaccines against SARS-CoV-2 have been granted an emergency use authorization, including BBIBP-CorV (Sinopharm) and CoronaVac (Sinovac) in China, the Pfizer-BioNTech COVID-19 vaccine (Pfizer) and the mRNA-1273 vaccine (Moderna) in the United States, and the Sputnik-V vaccine in Russia. Additionally, the protective rates and effectiveness of each vaccine are over 70% [81,82,83,84,85] against the early pandemic SARS-CoV-2 strains as well as the recently circulating strains, suggesting the disease can be controlled by vaccination in the future (Table 2).

Notably, most of these vaccines, except inactivated vaccines, are targeted at the viral spike or its RBD. Whether these vaccines are still effective against the SARS-CoV-2 mutants that continue to be produced in the population is still controversial. For example, Shi et al. found that human sera from a BTN162b2 (a nucleoside-modified RNA vaccine that encodes the SARS-CoV-2 full-length)-vaccinated person can neutralize three mutant viruses, including the N501Y strain from the UK and South Africa, the 69/70-deletion + N501Y + D614G strain from the UK, and the E484K + N501Y + D614G strain from South Africa [82]. Although antibodies, such as Regeneron’s REGN-COV2 cocktail and Eli Lilly’s LY-CoV016 antibody, can effectively neutralize the virus, viral mutations in the prevalent strains can weaken the recognition mediated by the monoclonal antibodies and polyclonal human serum [77,78]. These results suggest SARS-CoV-2 may escape the human immune response via continuous evolution by substitution in the RBD and/or deletion and insertion in the N-terminal domain loops of the spike, especially in an immunocompromised host [77,78]. Moreover, two recent reports showed that it is not a wise strategy to acquire herd immunity through natural infection [86,87]. In particular, antibody-dependent enhancement (ADE) was induced in the patients in an NTD-dependent manner, which can promote conformational changes of the viral RBD and, thus, enhance the binding of the viral spike to the cellular receptor as well as the virus infectivity [87]. Moreover, as predicted by the experts, SARS-CoV-2 may become similar to other human coronaviruses causing the common cold and coexist with humans [88]. Therefore, we strongly recommend developing multivalent vaccines targeting different epitopes of the RBD and conserved viral components, such as nucleocapsid, envelope, and membrane proteins, as soon as possible to control the current pandemic and prevent the emerging pandemic.

The identification of cross-reactive antibody epitopes in the viral genome can provide information for rational design strategies of vaccines and treatments for a variety of highly pathogenic coronaviruses, which is valuable for current and potential future epidemics. As reported, naïve antibodies expressed by the naïve B cell can neutralize SARS-CoV, SARS-like WIV1-CoV, SARS-CoV-2 and its mutants via recognition of the viral receptor-binding domain [89]. Cross-reactive epitopes are mainly located in the viral S, N, and ORF1ab, among which the S2 subunit of the spike protein contains the most promising cross-reactive epitopes, but the immune response induced by ORF1ab epitopes shows high individual specificity [90]. Six monoclonal antibodies that cross-reacted with the S proteins of SARS-CoV, SARS-CoV-2, and other coronaviruses via diverse epitopes were identified [91]. Human monoclonal antibody (mAb) 47D11 can specifically recognize the downward conformation of the viral RBD by targeting the conserved hydrophobic pocket in the RBD of SARS-CoV and SARS-CoV-2, and thus capturing these two viruses in a completely closed and a partially open conformation, respectively [92]. The neutralization efficiency of 47D11 is not affected by the mutations in the RBM (receptor binding motif), such as K417N, E484K, or N501Y, which makes 47D11 a promising therapeutic candidate against the new and rapidly spreading SARS-CoV-2 and its variants [92]. Further research shows that 47D11 can effectively neutralize ACE2-dependent betacoronaviruses, such as SARS-CoV, SARS-CoV-2, and the SARS-like bat betacoronavirus, WIV16 [92]. RBD trimer adjuvanted with Alum-3M-052 can induce effective neutralizing antibodies and protect mice and rhesus monkeys from SARS-CoV-2 infection [93]. A cocktail homotrimeric RBD based on different sarbecoviruses can induce effective cross-neutralizing responses to pan-sarbecovirus by interacting with the RBD epitopes outside of the RBM [94]. Chimeric spike mRNAs adjuvanted with lipid nanoparticle (LNP) can induce robust protective neutralizing antibodies against high-risk heterologous sarbecoviruses [95]. Furthermore, mosaic-RBD-nanoparticles elicited high-level antibodies with a superior cross-reactive recognition of heterologous RBDs compared to that of homotypic nanoparticles or COVID-19 convalescent human plasmas [96]. The neutralization activity of multivalent nanoparticles targeting different epitopes on the RBD is more than 100 times higher than that of monovalent nanoparticles [97]. The Spike-Ferritin nanoparticle (SpFN) and RBD-Ferritin nanoparticle (RFN), which use ferritin as a scaffold for nanoparticle synthesis, can stimulate a neutralizing titer more than 20-fold higher than that of the convalescent serum and protect the humanized ACE2 transgenic mice from virus challenge by blocking viruses (SARS-CoV-2 and its variants, as well as SARS-CoV) binding to ACE2 [98]. Moreover, the combination of two antibodies, A23–58.1 and B1-182.1, isolated from convalescent donors, exhibit high neutralizing activities against 23 VOCs (IC_50_ <0.6–28.3 ng/mL), including the B.1.1.7, B.1.351, P.1, B.1.429, B.1.526, and B.1.617 VOCs, by binding to a spike with all the RBDs in the up position [99]. These results indicate that the heterologous multi-target vaccines, such as chimeric spikes and neutralizing nanoparticles [96,97,100], can effectively inhibit SARS-CoV-2 infection and other coronaviruses, which is a promising vaccine against pan-coronaviruses.

Another strategy is to identify cross-reactive CD4^+^ and CD8^+^ T cell epitopes, which are highly conserved in human and animal coronaviruses, as the immune targets of the pan-coronaviruses vaccines [79,89,101,102]. Using immuno-informatics and sequence alignments, Prakash and colleagues identified 27 highly conserved potential human CD8^+^ T cell epitopes and 16 potential CD4^+^ T cell epitopes, which are conserved in the genome of SARS-CoV-2 and its circulating variants, the four major common cold coronaviruses (hCoV-OC43, hCoV-229E, hCoV-HKU1, and hCoV-NL63), and several SARS-like-CoV strains from bats and pangolins [79]. The replicase polyprotein ORF1ab appeared to be the most immunodominant Ag to stimulate cross-reactive CD4^+^ and CD8^+^ T cells and recall specific memory CD4^+^ T and CD8^+^ T cells, followed by the spike glycoprotein [79]. The memory CD4^+^ T cells stimulated by intranasal vaccination in the airway have strong protection against human coronaviruses [101]. Furthermore, mosaic antigens based on the conserved cytotoxic T lymphocyte were designed for pan-coronaviruses, including HCoV-NL63, HCoV-229E, HCoV-OC43, HCoV-HKU1, SARS-CoV, MERS-CoV, and SARS-CoV-2 [102]. Thus, multiepitope pan-coronavirus vaccines that include highly conserved B and CD4^+^ and CD8^+^ T cell epitopes of human-, bat-, pangolin-, and SARS-like-CoVs may be effective to protect against the current pandemic and future emerging coronaviruses.

## 4. Conclusions and Perspectives

At present, the COVID-19 pandemic is continuing all over the world, especially the second or third outbreak that occurred in some countries, such as India, Thailand, and Vietnam. As of 2 July, 2021, there were more than 182,319,261 confirmed cases, 3,954,324 deaths, and 2,950,104,812 vaccinations. However, with the frequent contact between humans and animals, and the continuous migration of wild animals, emerging coronaviruses will spillover from animals to humans, and the number of mutant viruses produced by recombination will also increase [1,4,5]. Therefore, it is urgent to design multi-target antiviral strategies to protect against emerging or re-emerging coronaviruses that appear or reappear at present or in the future.

By using high-throughput genome-scale screenings, such as phage display library, CRISPR-based library, and electro-chemiluminescence-based multiplex assay [57,90,103,104], it is hopeful that we will identify conserved cross-reactive epitopes or domains in coronaviruses and host proteins, which can be used for pan-coronavirus antiviral strategies, and design small-molecule drugs and peptides or identify existing pharmaceuticals and herbal medicines specifically targeting coronaviruses infection, such as viral spike protein-receptor binding and fusion, viral polymerase, nonstructural protein, and proteases. Moreover, other antiviral strategies are also required for pan-coronavirus inhibition. For example, the CRISPR-based antiviral strategy reported by Abbott et al. can effectively inhibit 90% of all known coronaviruses by degrading viral RNA with six CRISPR RNAs (crRNAs) targeting conserved viral regions [103]. Notably, vaccines are mainly used for healthy people to prevent infection, while medicines can be used for prevention and treatment. Antivirals, such as amantadine and interferon, are good examples of universal inhibitors for preventing and controlling viral infections. Therefore, scientists should give priority to the design, screening, and development of pan-coronavirus inhibitors, which are easier to realize and bring benefits to people faster at the early stage of infection.

## Figures and Tables

**Table 1:** Summary of the targets for pan-coronavirus inhibitor.

Target		Conserved Structure, and Infection or Pathogenic Mechanism	Strategies for Inhibiting Infection	Clinical or Experimental Stage of Promising Inhibitors	Reference(s)
Binding and fusion	*6-HB, HR1, and HR2*	Highly conserved 6-HB structure composed of HR1 and HR2 trimers is essential for membrane fusion and entry of coronaviruses.	Lipopeptides interact with HR1 and thus block the 6-HB formation.	In vitro, IC_50_ of EK1C4 is 4.2–187.6 nM. EC_50_ of Clofazimine is 1.25–5 μM.	[15,17,20,21]
	*Calcium channel*	Ca2+-mediated membrane fusion is critical for virus entry. Calcium cations interact with viral FP1 and FP2, resulting in membrane ordering.	Natural products bis-benzylisoquinoline alkaloids, such as neferine, can block host calcium channels.	In vitro, with median EC_50_ of 0.13–0.41 μM.	[22,23,24,25]
NSPs	*Viral proteases*	Viral PL^pro^ and 3CL^pro^ are involved in various steps in the virus infection, including cleavage of the polypeptides and inhibiting host immune responses, and posttranslational modification of host protein.	Inhibitors can bind conserved substrate-binding pocket of Mpro or PLpro. Raising endosomal pH to interfere with virus entry.	Bepridil EC_50_ is 0.86–0.46 μM against SARS-CoV-2, while it can reach a state C_max_ of 3.72 μM in vivo. ML188 inhibits SARS1 and SARS2 with IC_50_ of 4.5 and 2.5 µM.	[13,18,26,27,28,29,30,31]
	*Replication/transcription complex*	The replisome consists of at least eight NSPs, including NSP7–10, 12–14, and 16. Programmatic translation frameshifting at −1 of the ORF1b is conservative in all coronaviruses and is necessary for the synthesis of viral RdRp and downstream viral NSPs. NSP14 acts as N7-MTase and 3’-to-5’ proofreading exoribonuclease for modifying viral mRNAs. NSP16 activated by NSP10 participates in immune evasion.	Interacting with viral RdRp on conserved residues. Inserting nucleotide analogs into viral nascent RNA, resulting in lethal mutagenesis of the virus genome or termination of the polymerase extension. Targeting conserved metal-binding motifs of RdRp. Drugs or siRNA interfere with the “frameshifting” and inhibit virus replication. Conserved pocket formed between β3 and β4 of viral NSP16, and exoribonuclease domain of NSP14 are pan-coronavirus targets.	Lycorine can efficiently inhibit MERS-CoV, SARS-CoV, and SARS-CoV−2 with IC_50_ of 2.123, 1.021, and 0.878 μM, respectively. Merafloxacin inhibits the PRF of β-CoVs with IC_50_ of about 4.3–39 μM.	[32,33,34,35,36,37,38,39,40,41,42,43,44,45,46,47,48,49,50]
	*NSP1*	Inhibiting cellular translation by interacting with ribosomal subunit and/or inducing degradation of the host mRNA. Interacting with the SL region of the viral 5′UTR, promoting the recognition and binding of viral RNA and host ribosome, thus enhancing virus infection.	Inhibiting or competitively binding residues R124, K125, K164, and H165 in NSP1. Suppressing the SL1 with siRNA or sgRNA.	Not available	[51,52,53,54]
Host proteins		Host antiviral proteins inhibit virus infection by direct or indirect interaction. TMEM41B is necessary for the infection of several CoVs. Coronaviruses require cholesterol for viral entry, pathological syncytia formation, and pathogenesis. Host protease and HMGB1 are necessary for entry, whereas the SWI/SNF chromatin-remodeling complex is pro-viral for coronaviruses. Cyclophilins and immunophilins interact with viral NSP1 to activate immune cells and immune responses, resulting in immunopathological damage.	Targeting the key host factors of lipid metabolism may be a potential therapeutic strategy for pan-coronavirus. 25HC inhibits coronaviruses by depleting membrane cholesterol. SWI/SNF complex can be used as a pan-coronavirus target. LY6E is an anti-pan-coronavirus molecule by disturbing the virus-cell membrane fusion. CspA can efficiently block the replication of CoVs of all genera.	IC_50_ of 25HC is 550 nM, 2.48 μM, and 1.22 μM for SARS-CoV-2, SARS-CoV, and MERS-CoV. LY6E is a constitutively expressed ISG. All tested CoVs were inhibited by CspA with IC_50_ of 2.3–25 μM.	[55,56,57,58,59,60,61,62,63,64,65]

**Table 2:** Summary of the targets for pan-coronavirus vaccines.

Target	Strategies for Vaccine Development	Clinical or Experimental Stage of Promising Vaccines or Therapeutic Antibodies	Reference(s)
Viral RBD residues and other proteins	Multivalent vaccines targeting different epitopes of the RBD and conserved viral components. Multi-target nanoparticles.	The mAb 47D11 neutralizes ACE2-dependent SARS-like viruses. Chimeric spike mRNAs neutralize high-risk CoVs via prime-boost and adjuvanted with LNP in mice.	[89,92,93,94,95,96,97,100],
Cross-reactive CD4^+^ and CD8^+^ T cell epitopes	Multiepitope vaccines that include highly conserved B and CD4+ and CD8+ T cell epitopes. ORF1ab is the most immunodominant Ag targeted by CD4+ T cells, whereas structural and nonstructural proteins are immunodominant Ags that are targeted by CD8+ T cells.	Not available	[79,89,101,102]

## Data Availability

No datasets were generated or analyzed during the current study.
